# Draft Genome Sequence of *Microbacterium* sp. Strain KKR3/1, an Antimicrobial-Substance-Producing Strain Isolated from Banana Shrimp (Atyopsis moluccensis)

**DOI:** 10.1128/mra.01242-21

**Published:** 2022-02-03

**Authors:** Yanina Delegan, Anna Shorokhova, Anton Zvonarev, Tatiana Abashina, Natalia Suzina

**Affiliations:** a Skryabin Institute of Biochemistry and Physiology of Microorganisms, Pushchino Scientific Center for Biological Research of the Russian Academy of Sciences, Pushchino, Moscow Region, Russian Federation; University of Arizona

## Abstract

The strain KKR3/1 (VKM Ac-2910) was isolated from the microflora of the lower intestinal tract of the banana shrimp (*Atyopsis moluccensis*). The genome of the KKR3/1 strain consists of seven contigs, with a total length of 3,651,331 bp. The *N*_50_ value is 2,445,836 bp, and the GC content is 68.1%.

## ANNOUNCEMENT

The study of the microbial diversity of shrimp guts is extremely important because of a constantly growing volume of commercial production of crustaceans and the need to increase the quantity and quality of seafood produced under artificial conditions (1). The gut microflora of the shrimp *Atyopsis moluccensis* is of particular interest due to the unique feeding strategy of this organism. The absence of a number of its own digestive enzymes suggests the presence of a large microbial diversity in the composition of its gut microbiome (2, 3).

The strain KKR3/1 (VKM Ac-2910) was isolated from the microflora of the lower intestinal tract of the banana shrimp (*Atyopsis moluccensis*). Material from the lower intestine of shrimp was obtained by surgical dissection. Then the material (∼1 mm^3^) was resuspended in phosphate buffer (pH 7.0) (2 mL) and plated on agar culture media of different compositions (4). The grown colonies were microscopically examined, and a bacterial isolate (strain KKR3/1) with an ultrasmall cell size was selected. The cells of the KKR3/1 strain are represented by ultrasmall short rods or ovoids with a cell size of 0.4 × 0.5 μm. For long-term storage, the strain was kept in glycerol (40%) stocks at −70°С. For short-term maintenance, the strain was cultured on LB agar plates at 27°C.

Genomic DNA was isolated from a fresh culture biomass (a colony) of *Microbacterium* sp. strain KKR3/1 grown on LB agar using the DNeasy blood and tissue kit (catalog number 69506; Qiagen, Germany). DNA samples were sequenced on a NovaSeq 6000 platform using the S2 reagent kit (2 × 100 bp) (catalog number 20012861; Illumina, USA). A paired-end library was prepared using the KAPA HyperPlus kit (Kapa Biosystems, USA). The quality control of reads was performed using FastQC (https://www.bioinformatics.babraham.ac.uk/projects/fastqc). We obtained 55,405,668 paired-end reads of <101 bp in size. Reads were analyzed with Trimmomatic v.0.39 (5) for adaptor removal. Reads with average Phred scores of ≥15 and lengths of >50 bp were *de novo* assembled using SPAdes v.3.15.0 (6). The quality of the assembled sequences was assessed using the QUAST v.5.0.2 tool (7). Coding DNA sequences (CDSs) were predicted and annotated using the NCBI Prokaryotic Genome Annotation Pipeline (PGAP) (8). The average nucleotide identity (ANI) value was calculated using the EzBioCloud web service (9). The percentage of digital DNA-DNA hybridization (DDH) was calculated using the Genome-to-Genome Distance Calculator v.2.1 (10). Default parameters were used for all software unless otherwise specified.

The genome of the KKR3/1 strain consists of seven contigs with a total length of 3,651,331 bp. The *N*_50_ value is 2,445,836 bp, the GC content is 68.1%, and the genome coverage is 968×. The total numbers of CDSs and RNAs were 3,502 and 53, respectively. Of the 3,502 CDSs, 1,874 genes (53.5%) were functionally annotated. Analysis of a fragment of the 16S rRNA gene was performed using BLAST (11). Based on the results of sequencing of the 16S rRNA gene (GenBank accession number OL826759), the species closest to the KKR3/1 strain is Microbacterium oxydans (GenBank accession number WAAP01000000). However, the ANI and DDH values with respect to the M. oxydans type strain were 84.45% and 52.40%, respectively; these values do not allow the strain to be unambiguously attributed to M. oxydans. Therefore, we define the KKR3/1 strain as *Microbacterium* sp.

In the genome of the strain, we identified genes involved in the biosynthesis of penicillins and cephalosporins, namely, isopenicillin-*N*-epimerase (EC 5.1.1.17), isopenicillin-*N*,*N*-acyltransferase (EC 2.3.1.164), β-lactamase class A (EC 3.5.2.6), and penicillin G amidase (EC 3.5.1.11).

### Data availability.

This genome project was deposited in GenBank under BioSample number SAMN24060600, BioProject number PRJNA789158, GenBank accession number JAJSDW000000000, and SRA accession number SRR17276339.
